# Complete Genome Sequence of Citrus Vein Enation Virus Identified from a Korean Yuja Tree

**DOI:** 10.1128/mra.00424-22

**Published:** 2022-08-02

**Authors:** Hee-Ji Yang, Eun Jin Choi, Su-Hyun Lee, Jeong-Hyun Woo, Seungmo Lim, Seong-Jin Lee

**Affiliations:** a Department of Plant Quarantine, Animal and Plant Quarantine Agency, Gimcheon, Republic of Korea; Portland State University

## Abstract

We determined the complete genome sequence of the citrus vein enation virus (CVEV) collected from a Korean yuja tree (Citrus junos). The CVEV genome has 5,983 nucleotides, showing 97.3 to 98.6% identity with complete genomic sequences of other CVEV isolates, with the highest similarity being to the isolate PCJ.

## ANNOUNCEMENT

Citrus vein enation virus (CVEV), a positive-sense single-stranded RNA virus belonging to the genus *Enamovirus* in the family *Solemoviridae*, has been reported in various *Citrus* species in several countries (1–3). In Korea, CVEV was first reported in 2017 and confirmed to be prevalent in *Citrus* spp. and Poncirus trifoliata (4, 5). To date, only three complete genome sequences of Korean CVEV isolates, one from Citrus unshiu and two from P. trifoliata trees, have been registered in GenBank (5).

In 2021, 30 leaf samples without obvious signs of disease were collected from a commercial yuja (Citrus junos) grove in Goheung-gun, Jeollanam-do, South Korea. Total RNA was extracted from each sample using the Maxwell RSC Plant RNA kit (Promega Corporation, Madison, WI, USA). Diagnosis of individual samples was performed using reverse transcription PCR (RT-PCR) with a CVEV-specific primer pair (VE16f/VE17r) (3). CVEV was detected in all the samples.

To determine the complete genomic sequence of CVEV obtained from the yuja tree in Korea, seven primer pairs were designed by aligning the complete genome sequences of CVEV isolates available in GenBank. One sample was selected, and RT-PCR was performed using the primer pairs listed in Table 1. The sequences of the 5′ and 3′ termini were determined using the 5′ rapid amplification of cDNA ends (RACE) system and the 3′ RACE system (Invitrogen, Carlsbad, CA, USA), respectively, with specific primers (Table 1). Each amplicon was cloned into the TA vector (RBC Bioscience, New Taipei City, Taiwan), and at least three clones of each amplicon were sequenced in both directions by using the Sanger method at Macrogen Inc., South Korea. Consensus sequences of each amplicon, that overlap each other, were assembled using SeqMan Pro 7.1.0 (DNASTAR, Madison, WI, USA) with default parameters.

**TABLE 1:** Primers used for the analysis of the complete genomic sequence of citrus vein enation virus obtained from a Korean yuja tree

Target region	Primer name	Sequence (5′ to 3′)	Position^*a*^	Expected size
ORF0	CVEV-1F	GACTCTCGGTTTCTCCTATAC	151–171	849 bp
	CVEV-1R	CTGTATCATAGCCCGACAG	999–981	
ORF1	CVEV-2F	CTCACAGCCAAGTGCTTGGA	832–851	1,022 bp
	CVEV-2R	GGAAGAACAACCGTGGCC	1853–1836	
ORF1	CVEV-3F	TGTGCCATGCATATTGGCCA	1789–1808	1,096 bp
	CVEV-3R	CCGTAGCTTTCCTGACAGGC	2884–2865	
ORF1 to ORF2	CVEV-4F	TTGGCCGAGCTTGTGAAGG	2539–2557	1,176 bp
	CVEV-4R	CTAGGCATCGAAGCCACTC	3714–3696	
ORF2 to ORF3	CVEV-5F	CGAAGAGCATAGCCTATCTTG	3508–3528	964 bp
	CVEV-5R	GTAAGGACGCATCTCTCCG	4471–4453	
ORF3 to ORF5	CVEV-6F	GTCCACGCAGAATGCGAAGA	4332–4351	809 bp
	CVEV-6R	AGGTTGCGCATGTACACGAT	5140–5121	
ORF5	CVEV-7F	CCGAGATGTGGAAGTGCGT	5020–5038	905 bp
	CVEV-7R	CTTCCTCATTTAATGACTGAGC	5924–5903	
5′ terminus	5RACE-1R	GCTAGCTCCCAACCAGTTAG	290–271	NA
	5RACE-2R	GTCGTAGGGGATGACATGGTAG	248–227	NA
3′ terminus	3RACE-1F	GGCGGTGATACGGTTTCTTA	5778–5797	NA

a Primer positions were based on the reference genome sequence of CVEV (isolate VE-1; GenBank accession number NC_021564).

Consequently, the complete genome sequence of the CVEV has 5,983 nucleotides (nt) and a GC content of 50.71% (GenBank accession number ON229074). The ORF finder tool (http://www.ncbi.nlm.nih.gov/orffinder/) with default parameters showed the occurrence of five open reading frames (ORFs) in the CVEV genome, the same as other CVEV isolates (1, 3). ORF0 (nt 219 to 1,283) encodes a putative silencing suppression protein (P0) and overlaps completely with ORF1. ORF1 (nt 208 to 2916) encodes a peptidase. A fusion protein (replicase polyprotein) produced by ORF1 and ORF2 was translated by a −1 frameshift (nt 208 to 2202 and nt 2202 to 4178). ORF3 (nt 4301 to 4876) encodes a coat protein. A fusion protein (aphid transmission protein) produced by ORF3 and ORF5 is translated by the read-through of the stop codon of ORF3 (nt 4301 to 5785).

The full-length genomic sequence of the CVEV was compared with all the complete genomes of CVEV isolates available in GenBank, which were collected on November 15th, 2021, using the Kimura 2-parameter model in the MEGA 11 software (https://www.megasoftware.net) with default parameters. The pairwise comparison showed that the CVEV genome shared 97.3 to 98.6% identity with other CVEV isolates. It had the highest sequence similarity to the Korean isolate, PCJ (accession number LC433634), identified in P. trifoliata.

### Data availability.

The complete genome sequence of the citrus vein enation virus determined in this study was deposited in GenBank under accession number ON229074.
